# Optimizing an Obesity Treatment Using the Multiphase Optimization Strategy Framework: Protocol for a Randomized Factorial Trial

**DOI:** 10.2196/19506

**Published:** 2021-01-18

**Authors:** Gary G Bennett, Dori Steinberg, Jamiyla Bolton, John A Gallis, Cayla Treadway, Sandy Askew, Melissa C Kay, Kathryn I Pollak, Elizabeth L Turner

**Affiliations:** 1 Duke Global Digital Health Science Center Duke University Durham, NC United States; 2 Department of Psychology and Neuroscience Duke University Durham, NC United States; 3 Duke University School of Nursing Duke University Durham, NC United States; 4 Duke Global Health Institute Duke University Durham, NC United States; 5 Department of Biostatistics and Bioinformatics Duke University Durham, NC United States; 6 Cancer Control and Population Sciences Duke Cancer Institute Durham, NC United States; 7 Department of Populations Health Sciences Duke University Durham, NC United States

**Keywords:** text message, digital health, weight loss, personalized

## Abstract

**Background:**

Effective weight loss interventions exist, yet few can be scaled up for wide dissemination. Further, none has been fully delivered via text message. We used the multiphase optimization strategy (MOST) to develop multicomponent interventions that consist only of active components, those that have been experimentally determined to impact the chosen outcome.

**Objective:**

The goal of this study is to optimize a standalone text messaging obesity intervention, Charge, using the MOST framework to experimentally determine which text messaging components produce a meaningful contribution to weight change at 6 months.

**Methods:**

We designed a 6-month, weight loss texting intervention based on our interactive obesity treatment approach (iOTA). Participants are randomized to one of 32 experimental conditions to test which standalone text messaging intervention components produce a meaningful contribution to weight change at 6 months.

**Results:**

The project was funded in February 2017; enrollment began in January 2018 and data collection was completed in June 2019. Data analysis is in progress and first results are expected to be submitted for publication in 2021.

**Conclusions:**

Full factorial trials are particularly efficient in terms of cost and logistics when leveraged for standalone digital treatments. Accordingly, MOST has the potential to promote the rapid advancement of digital health treatments. Subject to positive findings, the intervention will be low cost, immediately scalable, and ready for dissemination. This will be of great potential use to the millions of Americans with obesity and the providers who treat them.

**Trial Registration:**

ClinicalTrials.gov NCT03254940; https://clinicaltrials.gov/ct2/show/NCT03254940

**International Registered Report Identifier (IRRID):**

RR1-10.2196/19506

## Introduction

The obesity epidemic continues, currently affecting more than one-third of all Americans [1,2], with dire consequences, including chronic disease, premature mortality, and significant costs to the US health care system. Efforts to reduce obesity include successful behavioral weight loss treatments with frequent interactions with a trained counselor and regular self-monitoring of diet and/or exercise [3-5]. These treatments produce 7% to 10% weight loss after 6 to 12 months [6,7]. However, the intensity of these treatments limits their widespread dissemination. A rapidly emerging evidence base supports the use of digital interventions for obesity treatment. Through a wide variety of intervention designs, digital interventions can produce clinically meaningful weight loss and reach a large range of populations [8].

Texting, one of the most long-standing mobile health (mHealth) approaches, is the most frequently performed activity on mobile phones and has nearly 90% population penetration, particularly among racial and ethnic minorities [9-11]. Despite these advantages, few studies have investigated the effectiveness of standalone texting interventions for weight loss and what components of text messaging enhance engagement. Rather, texting is often used as one of several intervention delivery channels in obesity treatments [12], combined with web and/or email delivery [13,14], paper materials and monthly coaching calls [15,16], mobile apps [17], social media support groups [18], and interactions with a dietitian or physician provider [19]. We know little about how to design texting interventions in a manner that maximizes both engagement and dissemination potential [20].

Moreover, texting has been used in a myriad of ways, making it challenging to extract best practices. For example, some interventions text participants bidirectionally [21] to facilitate self-monitoring, while others text unilaterally to deliver tips and/or feedback. Interventions use a variety of frequencies, sending texts weekly, daily, or even multiple times a day [13]. Similarly, when tailored feedback is provided via text, it has been variably designed as a summary score of participant progress, links to graphical figures, or as text describing weight loss progress. These design choices are not trivial and may differentially affect intervention engagement, the most important predictor of weight loss outcomes [20].

Standalone digital interventions (eg, without human counseling) have the potential for substantial population-level impact, given their broad reach and low marginal costs of operation [22]. However, relatively few trials have tested digital interventions that are deployed in a fully standalone manner; most evidence-based digital treatments include support from a human interventionist [8]. There is limited evidence detailing the efficacy of standalone digital treatments, but they most often suffer from low user engagement and high rates of nonuse attrition [23]. Thus, a key challenge for standalone digital interventions is designing technologies that people will use long enough to experience positive benefits. Standalone interventions do not benefit from the type of accountability that a human interventionist can produce [24,25].

We sought to design a standalone, 6-month texting intervention that would retain maximal dissemination potential. Given the limited consensus in the empirical literature regarding how to best design a texting intervention, we leveraged the multiphase optimization strategy (MOST) [26] to aid in developing an optimized digital treatment package. Behavioral interventions are usually *packages* of intervention components. However, traditional trial designs (eg, two-arm randomized controlled trials) cannot determine which intervention components contribute most to weight loss, which might have limited effects or even detrimental effects. This limits our ability to create lean, cost-efficient interventions that can optimally affect clinical outcomes.

MOST offers a framework to help build efficient multicomponent treatment packages that contain only meaningful intervention components. Its first phase involves using theory to identify tenable intervention components. While behavioral science theory can help us develop interventions that produce weight loss, it is less useful in informing the design of technologies that individuals will want to use daily for the months needed to produce weight loss. Thus, we leveraged the Technology Acceptance Model (TAM), a dominant information services theory [27,28], which argues that people will be more likely to use technologies that help them achieve their goals (ie, perceived usefulness) and are easy to use. One tenet of the TAM is that if these two conditions are met, people will have positive attitudes toward use, which can increase technology use intentions. TAM argues that when technologies are easy to use, people will have higher self-efficacy for their use. Though this model is widely studied, few studies have used it to inform mHealth intervention design. The second phase of the MOST framework—the basis of this study—involves implementing an experimental trial to identify active intervention components that might be included in an optimized treatment package. The third phase of the MOST framework involves testing the optimized treatment package in a fully powered randomized clinical trial.

The aim of this paper is to present the design of our MOST optimization trial, Charge. The goal of the trial is to identify intervention components and levels that might be included in a 6-month, standalone texting treatment aimed at physical activity and diet changes that result in clinically meaningful weight loss (ie, >5% from baseline) at 6 months and weight loss maintenance at 12 months. We will also explore associations of engagement and nonuse attrition with weight change. The following section describes the study design and intervention.

## Methods

### Overview

From our comprehensive review of intervention trials utilizing text messaging for weight loss, we abstracted intervention design details. There was great similarity in the core interventions utilized; most used social cognitive theoretical approaches with an emphasis on self-efficacy as a primary mediator [12,14,15,17-19]. From an intervention design perspective, most interventions involved sending texts bidirectionally, using texting for self-monitoring, and providing tailored feedback. Thus, we did not test these components experimentally, but built them into our core intervention. However, there was marked variability in most other design components: frequency of texting, whether self-monitoring data were collected, referent tracking day, timing of monitoring, feedback scheduling, and use of skills training. From this group of components and guided by the TAM, we identified components that would heighten the perceived usefulness and perceived ease of use of our texting intervention. MOST also recommends that we design our intervention for real-world implementation. For example, the ongoing MOST trial by Bonnie Spring and Linda Collins is designed to find the most effective weight loss intervention that can be delivered for US $500 per person [29]. They acknowledge that they might not be able to achieve maximal weight loss at this cost, but they view US $500 as an optimal target for dissemination.

In selecting experimental conditions, we did not include components that might maximize weight loss (eg, individual or group counseling) or components that cannot be delivered via texting (eg, web-based materials, email feedback, automated phone calls, and apps). We recognize that these design choices might limit the magnitude of our treatment outcomes, relative to gold-standard treatments. However, we think these concessions will help us achieve the goal of creating a standalone texting intervention that can be optimally disseminated. Based on preliminary data collected as part of Phase 1 of this trial, we identified five intervention components (see Table 1) that can enhance perceived usefulness or ease of use.

**Table 1:** Reference and comparator levels for intervention components.

Intervention component	Reference level	Comparator level
Motivational message source	Expert-generated	Self-generated
Texting frequency	Weekly	Daily
Reminder timing	One	Multiple
Feedback level	Individual goal	Summary score
Performance comparison	Self	Others (group)

### Motivational Messaging Source: Self-Generated Versus Expert-Generated

Interventions frequently use text messages to enhance and sustain participant motivation. For these messages to enhance participant efficacy, they must be perceived as salient to the individual, be relevant to one’s personal circumstances, and incorporate familiar social norms, otherwise there is a risk of undermining efficacy [30]. Presently, most motivational messages are developed by content experts, who are guided by theory, evidence, and experience [14-19,31-34]. One possible way to increase the potency of motivational messaging is to change the source of the messages. Self-generated text messages may result in greater attitudinal and behavioral change because they (1) exemplify *optimal* tailoring (ie, one knows oneself best), (2) are seen as highly self-relevant, (3) are viewed as credible, (4) are more often remembered (ie, self-referring effect), and (5) generate less resistance to persuasive attempts than messages originated by others. This is consistent with Self-Determination Theory’s emphasis on increasing autonomous self-regulation and perceived competence [35]. Participants randomized to the self-generated condition will create text messages in two areas at baseline: reasons and competence for weight loss. The former will target autonomous self-regulation (ie, change stems from within), while the latter will target efficacy for weight-related behavior change. We will send texts from these pools randomly. We hypothesize that receipt of self-generated motivational messages, compared to expert-generated messages, will enhance intervention engagement, resulting in larger weight loss outcomes.

### Texting Frequency: Daily Versus Weekly

There is substantial variability in the number of days that texting interventions request self-monitoring data. Some studies have participants self-monitor daily [13-16,33], 3 to 5 times per week [18,19], and weekly [32,33]. Regular self-monitoring is believed to activate self-regulatory processes that result in energy restriction and increased physical activity. For optimal self-regulation, self-monitoring should be routine and feedback should be provided. Although daily self-monitoring might appear to be optimal, if the texts are perceived as burdensome, there is potential for nonuse attrition. Indeed, in one of our previous texting interventions, we found that engagement with our daily self-monitoring texts was high through 6 weeks but subsequently decreased [13]. By trial end, 49% of the participants texted daily. A somewhat less regular, but still routine, frequency (eg, weekly) might minimize perceived burden. Ours will be the first study to look at this question experimentally. We hypothesize that daily self-monitoring will be superior to once-weekly self-monitoring for maximizing intervention engagement and weight loss.

### One Reminder Versus Multiple Reminders

As noted, sustained intervention engagement is a key goal for standalone digital interventions. Thus, reminders might serve as a powerful aid to promote regular self-monitoring, the primary participant engagement in this study’s intervention. However, as with texting frequency, there may be concern that sending multiple self-monitoring reminders might promote user burden and intervention fatigue, thus negatively impacting engagement behaviors. The impact of reminder notifications on user behavior is largely unclear. There is limited empirical evidence that multiple reminders produce user burden; in fact, frequent notifications are often used in commercial digital interventions to enhance engagement. We randomized participants to receive either a single reminder or multiple reminders if they fail to self-monitor on their assigned day. We hypothesize that receiving multiple reminders will result in superior intervention engagement and weight loss outcomes.

### Feedback From Summary Scores Versus Individual Goals

Weight loss interventions in general, and our intervention specifically, implicitly seek to promote change in multiple behaviors simultaneously. Accordingly, self-monitoring interfaces, like ours, are designed to allow participants to track multiple behaviors in a given self-monitoring session. A challenge emerges when deciding how to provide feedback in response to self-monitoring. Feedback might describe performance singly for each tracked goal. Doing so best allows feedback to be tailored to a specific goal, which might be important given the potential for variability in user adherence to their assigned goals. In contrast, one might leverage recommendations from the risk communication literature, which describes the potentially superior performance of a single summary score that collapses across the variability in an individual’s assigned goals. Such a summary score might minimize the literacy and numeracy challenges in interpreting feedback, perhaps at the expense of offering the detailed feedback that might be necessary to promote change in discrete behaviors. We randomized participants to either the summary score group or the individual goal feedback group. We hypothesize that the summary score approach will facilitate easier processing and comprehension, thus improving engagement and weight loss outcomes.

### Self-Performance Versus Group Performance Comparison

Tailored feedback often benchmarks a participant’s performance against one’s own prior performance. An alternative is to use principles of gamification, comparing a user’s behavior change progress against that of other participants [36]. Such a *leaderboard* strategy can amplify competition and promote positive engagement and behavior change outcomes [37]. However, a potential risk of the leaderboard approach is that it seems to perform best when there are small relative differences between users; when users significantly underperform compared to their peers, the leaderboard approach might be demotivational [38]. We randomized participants to receive either comparison to themselves or comparison to their peers. We hypothesize that those who receive feedback benchmarked relative to their peers will have significantly better weight loss outcomes than those whose behaviors are compared to their own performance.

### Intervention Design

All participants will receive a core, 6-month, weight loss texting intervention built on a theory-based approach. Participants will be assigned an individualized set of routine lifestyle behavior change goals and directed to make small behavioral changes to create an energy deficit sufficient to produce weight change. We developed this approach and named it the interactive obesity treatment approach (iOTA). We designed iOTA specifically for delivery via digital health methods, such as texting, where sustained intervention engagement is critical. Individuals expect their digital health experience to be straightforward and highly personalized with minimal effort. Accordingly, iOTA does not require expert knowledge or expensive resources [13,39]. From the participants’ perspective, they simply take a short survey and are immediately assigned a set of personally tailored behavior change goals. Participants use text messaging to self-monitor their adherence to these goals. They receive tailored feedback based on their progress, skills-training videos, and motivational texts.

All core iOTA intervention components are designed to target self-efficacy, which we selected from Social Cognitive Theory [40,41], given its consistent association with weight loss outcomes [42-44]. Bandura identified four primary factors [45] that influence self-efficacy: mastery experiences, social modeling, social persuasion, and somatic and emotional reactions. Our text messages will cover all of these domains. Social Cognitive Theory also indicates that behavior change can be facilitated through a number of self-regulatory processes, including self-monitoring [46-48], goal setting [24,44], and social support [49,50].

### Obesogenic Behavior Change Goals

Each participant will track four behavior change goals that produce an energy deficit sufficient to produce weight loss. We have a library containing obesogenic behavior change goals that have been selected based on their (1) empirical support, (2) population relevance, and (3) ease of self-monitoring. We adapted the goal library that was deemed efficacious in our previous studies [22,31]. All participants take a short survey—the iOTA survey—and are immediately assigned a set of personally tailored behavior change goals. Our prescription algorithm assigns three tailored behavior change goals and one universal goal for each 8-week goal cycle (see Textbox 1). The prescription algorithm prioritizes behaviors based on highest need of change, those for which the participant has high self-efficacy and readiness, and those that achieve the intended caloric deficit.

**Behavior change goals.**

***Universal goal assignment:***

- Cycle 1: no red zone foods
- Cycle 2: portion control
- Cycle 3: walk 10,000 steps per day

***Goal list (in cycle date order):***

- Goal 1: no sugary drinks
- Goal 2: no sweet snacks
- Goal 3: no fast food
- Goal 4: no fried food
- Goal 5: 5+ fruits and vegetables
- Goal 6: no salty snacks
- Goal 7: fast between 7 PM and 7 AM
- Goal 8: eat grains and starches <1× per day
- Goal 9: no red meat
- Goal 10: restaurants ≤1× per week
- Goal 11: ≤2 hours of TV per day
- Goal 12: ≤1 alcoholic drink per day
- Goal 13: get brisk activity
- Goal 14: do strength training 2× per week
- Goal 15: no high-fat seasoning

### Self-Monitoring and Tailored Feedback

Regular self-monitoring is a robust predictor of weight loss, although adherence typically wanes over time [46,48,51]. Disengagement likely results from usability limitations, cognitive complexity, and lack of immediate feedback. As a result, our trials have involved extensive testing to ensure that our texting self-monitoring tools are engaging. Our text messaging system is fully automated, currently operational, uses open source technologies, and is designed for scalability.

Depending on their randomization status, we will contact participants either daily or weekly. An outbound text will request self-monitoring data (ie, a prompt) based on a participant’s behavior change goals. For example, the system may ask if an individual walked 10,000 steps yesterday. Participants will then respond by text to the prompt with their self-monitoring data. We immediately provide tailored, real-time feedback on participants’ progress via text. The feedback an individual receives depends on their randomization: whether they are being compared to themselves or the group and whether they receive feedback from summary scores or from each individual goal. We will also provide skills-training tips, tailored to each participant’s assigned goals.

### Tailored Skills-Training Videos

We have skills-training videos, 2 to 5 minutes in length, for each of the goals in our library. For example, for those assigned a fast food–reduction goal, we will provide skills-training materials on eating out, social eating, and lunch packing. We will also have a larger library of general behavior change skills (eg, stimulus control, problem solving, social cues, and stress management). Our materials include tailored narratives and information about cost and community resources. We sent links to videos at the beginning of each goal period that correspond to participants’ goals that are assigned as discussed in the *Obesogenic Behavior Change Goals* section above.

### Participants

Participants are adult men and women, aged 18 to 65 years, who have English-language proficiency and a BMI above 25 kg/m^2^. We aim to recruit a sample of 448 participants that is 30% male and 40% racial or ethnic minority, similar to the demographics of Durham, North Carolina, United States. Participants are required to own a smartphone and be willing to receive multiple text messages daily. Exclusion criteria include the following: prior or planned bariatric surgery; psychiatric hospitalization in the past 12 months; pregnant, nursing, or planned pregnancy; history of a cardiovascular event; history of an eating disorder; history of a health condition (eg, end-stage renal disease, cancer, or schizophrenia) or use of medications (eg, lithium, steroids, or antipsychotics) that would affect weight measurement, for which weight loss is contraindicated, or might promote weight change; current participation in a weight loss trial and/or recent weight loss of more than 10%; and investigator discretion, for safety reasons.

### Recruitment and Screening

Participants were recruited using the following multipronged strategy: (1) direct marketing, (2) local media, (3) social media, (4) snowball recruitment, and (5) community organizations. We have used these methods successfully in many of our team’s previous studies. The geographically targeted postings were placed on Nextdoor, Facebook, and Reddit.

Those who responded to initial study marketing were directed to a preliminary eligibility screening assessment via a Qualtrics online survey. If deemed eligible via the screening assessment, participants were then invited to complete the study’s online informed consent process and baseline surveys. Those who completed all surveys were then invited for an in-person visit to confirm participant eligibility. At the baseline visit, study staff collected written informed consent and confirmed participant eligibility by taking anthropometric measurements. After randomization, each participant received an individual orientation to the intervention.

### Procedures

All study procedures were registered at ClinicalTrials.gov (NCT03254940). After confirming eligibility, participants were randomized to one of 32 experimental conditions (see Multimedia Appendix 1) to test the intervention components, including frequency (weekly vs daily), motivational messaging (self- vs expert-generated), reminders (one vs multiple), feedback type (summary score vs individual score), and comparison unit (self vs group). We randomized participants using a permuted block method with stratification for gender. A computer algorithm sorted each participant into three self-reported gender categories—male, female, or any other self-description—and then assigned them accordingly into blocks of the 32 groups, randomly ordered by the computer. The computer algorithm, developed by a software engineer as directed by a biostatistician, minimized selection bias by generating the blocks ad hoc, thereby preventing prediction of the assignment order. Due to the logistics of enrollment and quality control of the intervention, it was impossible to completely blind the data collection staff to treatment assignment. However, all possible steps were taken to reduce unnecessary awareness of treatment assignment and to limit opportunities for the introduction of bias into the data by our data collection staff. We will analyze outcomes while blinded to allocation status.

### Data Collection

#### Survey Data

Data were collected at four study visits: baseline and 3, 6, and 12 months postbaseline. A protocol window for follow-up assessments was defined as 2 weeks before to 4 weeks after the 3-, 6-, and 12-month dates, relative to the baseline visit. Every reasonable effort was made to complete an in-person follow-up visit within the protocol window, including multiple rescheduling attempts and reminders. During the last week of the protocol window, we approached participants who refused or were unable to attend an in-person follow-up visit using a secondary data collection protocol. We emailed participants a link allowing them to access the online survey assessments on their own and asked them to provide a weight photograph or self-reported weight.

Surveys were administered in English via computer using an online survey tool where questions required responses to ensure completeness. Demographic variables collected at baseline included age, gender, race or ethnicity, marital status, parity, height and weight, socioeconomic status, insurance status, occupational status, and educational attainment.

#### Primary Outcome

The primary outcome is weight in kilograms. During in-person visits, participants removed shoes and items from pockets prior to height and weight measurements. Height was measured to the nearest 0.1 cm using a calibrated wall-mounted stadiometer [52]. Body weight was measured to the nearest 0.1 kg using a portable electronic scale [52]. BMI was calculated using participant height collected at baseline and weights collected at sequential in-person visits or self-reported weights if the participant was unable to come in person.

#### Secondary Outcomes

##### Physical Activity Outcomes

Physical activity was measured using the Global Physical Activity Questionnaire version 2 (GPAQ 2) at baseline and at 6 months postrandomization [53]. The GPAQ 2 was developed by the World Health Organization to measure physical activity participation as well as sedentary behavior. Participants were asked to report the frequency and duration of moderate to vigorous physical activity participation at work, travel to and from places and recreational activities, as well as the time spent being sedentary, in a typical week.

##### 12-Item Short Form Health Survey Outcomes

Adapted from the 36-item Short Form Health Survey (SF-36) [54], the 12-item Short Form Health Survey (SF-12) [55] has been operationalized for large-scale health measurement and monitoring efforts for all age groups. Participants were asked to complete the survey at baseline and at 6 and 12 months postrandomization to understand mental (ie, mental health, role emotional, and social functioning scales) and physical (ie, physical functioning, role physical, or bodily pain scales) performance and overall health-related quality of life. The age-specific mean difference score will be calculated from a participant’s physical and mental health composite score to compare participants to their age group’s mean score.

##### Engagement Outcomes

Intervention engagement is defined as successful self-monitoring during an expected window of time after a self-monitoring prompt was sent to the participant via text message. The window of time and frequency of the prompts delivered is determined by participant allocation to the five components of the intervention across the 6-month intervention period. Self-monitoring is considered complete if the participant texted back a properly formatted response with self-monitoring data for their four behavior change goals. For each participant, we will calculate a rate of completed responses out of the number expected.

### Statistics

#### Overview

Using the FactorialPowerPlan macro from SAS 9.4 (SAS Institute) [56], developed by the Penn State Methodology Center, University Park, Pennsylvania, we computed the sample size needed for a full 2^5^ factorial design. We determined that a total sample size of 282 participants would be required to achieve an overall power of 80% to detect a 0.75-kg difference in weight loss. We conservatively assumed 30% dropout at 6 months, so we inflated the sample size to 403. To obtain an equal number of participants in each treatment combination (ie, condition), we further inflated this sample size to 448, which would give a final sample of 14 participants in each of the 32 conditions. To achieve greater gender and racial diversity consistent with our recruitment goals, enrollment may be amended to perform additional targeted recruitment.

#### Statistical Analysis

For each of the five components, participants’ weights and demographics will be summarized by levels of the component. Continuous variables will be summarized using means, standard deviations, medians, quartiles, and ranges, and categorical variables will be summarized using counts and percentages.

In the primary analysis, those whose data were collected outside of the study window or who sent in weight measured at home, rather than having weight measured in person, will be treated as missing for that time point. Sensitivity analyses will be performed including such data.

The primary outcome is absolute change in weight from baseline to 6 months. We will estimate the main effects of each intervention component on the primary outcome and of all pairwise, three-way, four-way, and five-way interactions of those components at each follow-up time point using a linear mixed-effects model. In order to estimate effects on weight change, we will model all four weights for each individual (ie, baseline and 3-, 6-, and 12-month follow-ups) in the same model. The model will include fixed effects for time point (ie, 3 months, 6 months, and 12 months); the time × component, time × pairwise, time × three-way, time × four-way, and time × five-way interactions; up to five-way interactions; and gender, the variable by which the randomization was stratified. We will not include the effects of the components and their interactions at baseline. This constrains the baseline comparisons to be equal, which is appropriate in a randomized trial and increases power [57,58]. We will model the correlation between repeated measures on the same individual using an unstructured residual covariance matrix. The model will allow us to estimate weight change and percentage weight change at the interim time point (ie, month 3) and final follow-up time point (ie, month 12), but the main estimates of interest will be the interaction of the five treatment indicators by month 6.

After examining the model, we will assemble a multicomponent intervention package. If a component has a main effect on weight loss at 6 months that is greater than or equal to 0.7 kg (1.5 lbs) and no significant interaction with another component, then the superior level of the component will be retained for the intervention package. Otherwise, if there is no significant main effect or interaction, the default (ie, reference) level of the component will be retained (see Table 1). We will reconsider inclusions based on the presence of large (ie, effect ≥0.7 kg) interactions. This decision making is based on the approach outlined in an article by Collins et al [59]. Although the 3- and 12-month weight will not be used in the primary decision-making process, we may reconsider our inclusions if there is a large change in effect between 6 and 12 months, or if there are larger effects at 3 months. Given the factorial design, we will use effect coding, rather than dummy coding, for analyzing the effects of the intervention components. If the sample size is equal per condition, all of the tests of main effects and interactions are uncorrelated; that is, the main effect of a condition is the same even if other treatment conditions and interactions are included in the statistical model. Even with unequal sample sizes across conditions, as may occur with differential dropout by condition, if the imbalance is minor, the correlations between effects should be small [26].

Missing outcome data due to dropout or missing intermediate visits are expected to be, at most, 30%. Since the mixed model will be fit using a full maximum likelihood method, we will be able to account for predictors of missingness in the model in order to obtain valid estimates of the main component effects, thanks to the property that the response can be missing at random [60]. In practice, we will compare baseline characteristics of completers and noncompleters. If we find that any covariates predict missingness, we will adjust for these variables in a sensitivity analysis model.

In addition, we will examine gender as a potential moderator of the intervention effects. We acknowledge that the study is not powered to detect moderators, but these results may be used to guide future studies.

## Results

The project was funded in February 2017; enrollment began in January 2018 and data collection was completed in June 2019. Data analysis is in progress and first results are expected to be submitted for publication in 2021.

## Discussion

### Overview

The intensive nature of most traditional weight loss interventions constrains their dissemination. To make a population-level impact in addressing the obesity epidemic, we need treatments that are novel, easy to disseminate, and sustainable. The overarching goal of Charge is to develop an efficacious, standalone, text messaging obesity intervention. We focus on standalone approaches—treatments that can be delivered solely via text messaging—because of their dissemination potential. Standalone treatments are scalable, affordable, and can achieve population reach. They can reach into broad, diverse, and geographically dispersed populations that do not have stable access to other approaches (eg, counseling, web, email, and video) frequently employed in digital weight loss trials. However, these interventions are also modular; they can be delivered independently to maximize cost-effectiveness or combined with other approaches (eg, provider counseling) to maximize outcomes.

### Comparison to Prior Work

Despite their translational potential, few trials have tested a standalone texting intervention for weight loss [8]. While the widespread availability of mHealth apps has been an undeniable boon for Americans who are seeking easy access to strategies to help them manage their health, very few of the commercially available digital solutions have any evidentiary basis. Further, engagement with apps wanes over time, making them a potentially less appealing approach [25,61]. Also, apps require data plans that are prohibitive for those on limited incomes [39]. In contrast, it has been extremely difficult to promote the translation of evidence-based treatments, both because of the latency in the process and because few research-tested digital treatments match the needs of the broader marketplace. Our interest is in developing an intervention solution that would be suitable for population distribution; accordingly, we designed it to match the conventions of the most widely accessible digital tools.

Charge is one of the first trials to apply the MOST framework to the development of a digital health intervention. For several reasons, MOST is particularly well suited to this task. There are myriad ways to design digital treatments, particularly those that are standalone. Indeed, design considerations and their impact on user behavior receive considerable attention in commercial software design. In contrast, the empirical literature provides little guidance on optimal intervention designs, and the extant evidence is replete with interventions with designs that vary considerably. This is problematic because even minor changes to the design of digital components can markedly impact user engagement, the most important predictor of weight loss outcomes. MOST allows us to isolate the weight loss effects of discrete texting intervention components and to then assemble an efficacious standalone texting treatment package. Of note, MOST encourages optimization for a specific purpose. Here we have chosen to optimize a standalone treatment for broad public health delivery, similar to the text4baby intervention [62]. What MOST describes as the continuous optimization principle could also be described as iteration, a concept that is fundamental to modern software design. In principle, when Charge is complete, one might continue the optimization process to continue refining our interventions for optimal effectiveness. Indeed, our group has plans to do this at the conclusion of the Charge trial.

### Limitations

There are some study limitations. One limitation is whether we selected the appropriate components, component levels, and number of components. As mentioned previously, we understand that these design choices might constrain the magnitude of our treatment outcomes, relative to gold-standard treatments. However, our goal is to create a standalone texting intervention that can be optimally disseminated. Additionally, lack of diversity in sampling could be a limitation. It is important for our sample to be diverse, particularly considering evidence that racial and ethnic minorities both experience the health effects of obesity at disproportionately high rates, and that the use of text messaging in these groups is almost ubiquitous [10]. We also acknowledge that we have made design decisions that may constrain efficacy and retention, including the lack of human contact with study participants. It is a common observation that standalone treatments have tremendous challenges with nonuse and trial attrition [25,61].

### Conclusions

Although many MOST screening experiments utilize factorial experimental designs, full factorial trials are particularly efficient in terms of cost and logistics when leveraged for standalone digital treatments. The marginal costs of adding users accrue primarily due to research costs and quite minimally due to intervention expenses. Accordingly, MOST has the potential to promote the rapid advancement of digital health treatments. This is particularly important for standalone treatments; despite mounting feasibility evidence, such interventions frequently report suboptimal weight loss outcomes. For these interventions to have population-level impact, we must make their outcomes more robust. Charge is designed to address this goal. Subject to positive findings here and in a future efficacy trial, the intervention will be low cost, immediately scalable, and ready for dissemination. This will be of great potential use to the millions of Americans with obesity and the providers who treat them.

## Appendix

Multimedia Appendix 1 Randomization conditions by group.

## Abbreviations

GPAQ 2: Global Physical Activity Questionnaire version 2
iOTA: interactive obesity treatment approach
mHealth: mobile health
MOST: multiphase optimization strategy
SF-12: 12-item Short Form Health Survey
SF-36: 36-item Short Form Health Survey
TAM: Technology Acceptance Model
