# Supplementary material for: Optimizing an Obesity Treatment Using the Multiphase Optimization Strategy Framework: Protocol for a Randomized Factorial Trial
Source: JMIR Res Protoc. 2021 Jan 18;10(1):e19506. doi: 10.2196/19506 (PMC7850907; doi:10.2196/19506)
Supplement: Multimedia Appendix 1 [file resprot_v10i1e19506_app1.docx]

**Appendix 1: Randomization Conditions by Group**

| Group # | Frequency | Motivational Messages | Reminders | Comparison Unit | Feedback Type |
| --- | --- | --- | --- | --- | --- |
| 1 | Daily | Expert | Single | Group | Summary |
| 2 | Daily | Expert | Single | Group | Individual |
| 3 | Daily | Expert | Single | Self | Summary |
| 4 | Daily | Expert | Single | Self | Individual |
| 5 | Daily | Expert | Multiple | Group | Summary |
| 6 | Daily | Expert | Multiple | Group | Individual |
| 7 | Daily | Expert | Multiple | Self | Summary |
| 8 | Daily | Expert | Multiple | Self | Individual |
| 9 | Daily | Self | Single | Group | Summary |
| 10 | Daily | Self | Single | Group | Individual |
| 11 | Daily | Self | Single | Self | Summary |
| 12 | Daily | Self | Single | Self | Individual |
| 13 | Daily | Self | Multiple | Group | Summary |
| 14 | Daily | Self | Multiple | Group | Individual |
| 15 | Daily | Self | Multiple | Self | Summary |
| 16 | Daily | Self | Multiple | Self | Individual |
| 17 | Weekly | Expert | Single | Group | Summary |
| 18 | Weekly | Expert | Single | Group | Individual |
| 19 | Weekly | Expert | Single | Self | Summary |
| 20 | Weekly | Expert | Single | Self | Individual |
| 21 | Weekly | Expert | Multiple | Group | Summary |
| 22 | Weekly | Expert | Multiple | Group | Individual |
| 23 | Weekly | Expert | Multiple | Self | Summary |
| 24 | Weekly | Expert | Multiple | Self | Individual |
| 25 | Weekly | Self | Single | Group | Summary |
| 26 | Weekly | Self | Single | Group | Individual |
| 27 | Weekly | Self | Single | Self | Summary |
| 28 | Weekly | Self | Single | Self | Individual |
| 29 | Weekly | Self | Multiple | Group | Summary |
| 30 | Weekly | Self | Multiple | Group | Individual |
| 31 | Weekly | Self | Multiple | Self | Summary |
| 32 | Weekly | Self | Multiple | Self | Individual |
